# Towards Personalised Mood Prediction and Explanation for Depression from Biophysical Data

**DOI:** 10.3390/s24010164

**Published:** 2023-12-27

**Authors:** Sobhan Chatterjee, Jyoti Mishra, Frederick Sundram, Partha Roop

**Affiliations:** 1 Department of Electrical, Computer and Software Engineering, Faculty of Engineering, University of Auckland, Auckland 1010, New Zealand; 2 Neural Engineering and Translation Labs, Department of Psychiatry, University of California, San Diego, CA 92093, USA; jymishra@health.ucsd.edu; 3 Department of Psychological Medicine, Faculty of Medical and Health Sciences, University of Auckland, Auckland 1023, New Zealand; f.sundram@auckland.ac.nz

**Keywords:** mood prediction, mood score, mood-state classification, depressive-mood prediction, wearable data, deep learning, explainable model, explainable AI, model optimisation

## Abstract

Digital health applications using Artificial Intelligence (AI) are a promising opportunity to address the widening gap between available resources and mental health needs globally. Increasingly, passively acquired data from wearables are augmented with carefully selected active data from depressed individuals to develop Machine Learning (ML) models of depression based on mood scores. However, most ML models are black box in nature, and hence the outputs are not explainable. Depression is also multimodal, and the reasons for depression may vary significantly between individuals. Explainable and personalised models will thus be beneficial to clinicians to determine the main features that lead to a decline in the mood state of a depressed individual, thus enabling suitable personalised therapy. This is currently lacking. Therefore, this study presents a methodology for developing personalised and accurate Deep Learning (DL)-based predictive mood models for depression, along with novel methods for identifying the key facets that lead to the exacerbation of depressive symptoms. We illustrate our approach by using an existing multimodal dataset containing longitudinal Ecological Momentary Assessments of depression, lifestyle data from wearables and neurocognitive assessments for 14 mild to moderately depressed participants over one month. We develop classification- and regression-based DL models to predict participants’ current mood scores—a discrete score given to a participant based on the severity of their depressive symptoms. The models are trained inside eight different evolutionary-algorithm-based optimisation schemes that optimise the model parameters for a maximum predictive performance. A five-fold cross-validation scheme is used to verify the DL model’s predictive performance against 10 classical ML-based models, with a model error as low as 6% for some participants. We use the best model from the optimisation process to extract indicators, using SHAP, ALE and Anchors from explainable AI literature to explain why certain predictions are made and how they affect mood. These feature insights can assist health professionals in incorporating personalised interventions into a depressed individual’s treatment regimen.

## 1. Introduction

Depression is a disorder involving a loss of pleasure or interest in activities for long periods and is associated with sustained mood deterioration [1]. It can affect several aspects of life, including relationships and work. According to the World Health Organisation (WHO) 2023 estimates, 5% of adults (approximately 300 million) worldwide experience depression, with women 50% more likely to experience depression than men. It is a significant contributor to the 700,000 suicides every year around the world [2]. Despite this, more than 75% of people in low- and middle-income countries receive no treatment due to a lack of investment in mental health, a lack of healthcare professionals and social stigma associated with mental health disorders [2]. For those who receive treatment, antidepressant medications are often the first line of treatment. However, they have a low effectiveness as only one-third of all patients show symptom remission, as evidenced in large clinical trials [3,4].

Therefore, interest has grown towards approaches that supplement clinical interventions. Studies have shown that lifestyle interventions, such as better sleep hygiene [5], practising mindfulness [6], physical activity interventions [7] and dietary interventions [8,9,10], have promise in managing depression [11]. Given the prevalence of devices with sensors that can be used to monitor lifestyle activities, such as smartphones and smartwatches, researchers are proposing using such devices to detect, monitor and manage depression [12]. The use of wearable technology to supplement clinical approaches is particularly appealing as it is unobtrusive, real time, often passive (requiring little or no active input by a depressed individual/patient), of finer granularity (more data in the same time period) and allows assessments to occur in the person’s usual environment [13].

As changes in mood and consistently low mood are often associated with depression, studies have tried to use mood as an indicator to monitor and predict the progression of depression. Previous studies have used data from various sensors on wearable devices to either detect or predict future changes in mood. They have used GPS location [14,15,16], phone- and app-usage patterns [17,18,19], voice and ambient noise [20] and motion sensor information [21]. An Ecological Momentary Assessment [22] has also been used to predict mood [23,24] in depressed individuals. These studies have focused primarily on using Machine Learning (ML) and its subtype Deep Learning (DL) models to develop predictive models owing to their excellent ability to learn associations in complex data. Moreover, other studies categorise the sensor data into activity data, sleep data, heart data or phone-usage data and then build ML- and DL-based predictive models by using them [21,25,26,27,28,29,30,31].

Nevertheless, most previous studies using ML- and DL-based predictive models have focussed on cross-sectional research, despite the failure of cross-sectional studies to apply to larger, more representative samples [32]. Moreover, cross-sectional works fail to account for the substantial interindividual variability in clinical response to the same treatment or behavioural recommendations for depression due to genetic, environmental, behavioural, lifestyle and interpersonal risk factors [33,34]. Personalised models built on longitudinal data are more suited to account for such variability. Therefore, recent works have begun focusing on personalised predictive models for depression [16,23,25,35].

Furthermore, predicting mood scores is often insufficient in a clinical setting. Most ML and DL approaches are black-box approaches, i.e., they do not show how they reached a prediction [36]. Without explaining why a model predicts a mood score, healthcare professionals cannot determine what insights the prediction contains [37]. These insights can then be used to check a model’s fidelity (whether the model predictions make sense) [38] and suggest interventions that help manage the symptoms in a personalised fashion.

Recent advances in explainable Artificial Intelligence (XAI) offer solutions to the problem of trustworthiness in ML and DL models. Explainable models (we use the terms explainability and interpretability interchangeably in this work [38]) such as Decision Trees [36] can be easily processed/simplified to explain their outputs [39]. However, their expressive power is limited by their size, and increasing their expressiveness decreases their interpretability. DL models can make more complex associations from multimodal data and yield better-performing models [37,40] but are not explainable [36]. With the availability of post hoc explainable methods, such as Shapley Additive Explanations (SHAP) [41] and Local Interpretable Model-agnostic Explanations (LIME) [42], explaining performant black-box DL models has become easier [36].

Studies such as [43,44,45] use explainability techniques on ML models to obtain insights into the model outputs. Moreover, recent works have begun exploring explainability in mental health settings [24,46,47,48,49]. However, the use of explainability has been limited to the extraction of the most influential model features/inputs using SHAP or LIME [50]. Despite the high expressive power of DL models, the suitability of personalised models for depressive-mood prediction and the utility of explainable AI in establishing trustworthiness, the use of explainables in personalised DL mood-score prediction is currently lacking in academic literature.

Therefore, this work developed a novel DL-based post hoc explainable framework for personalised mood-score prediction. The models can be used to predict current mood scores from current biophysical signals and explain how patients’ activities affect their mood scores, suggesting possible indicators upon which to intervene for healthcare professionals and patients (for self-management). We illustrate our approach by using an existing multimodal dataset (from [24]) containing longitudinal Ecological Momentary Assessments (EMAs) of depression, data from wearables and neurocognitive sampling synchronised with electroencephalography for 14 mild to moderately depressed participants over one month. The work in [24] established the possibility of applying Machine Learning to a multimodal depression dataset with personalised prediction. We significantly extend that work by making three main contributions:

- A parallelised DL modelling and optimisation framework is proposed that helps train and compare multiple Multilayer Perceptron (MLP) DL models to predict participants’ mood scores=—a discrete score used to assess the severity of patients’ depressive symptoms. The MLP framework exceeds the performance of 10 classical ML models.
- Multiple post hoc explainable methods [36] are combined to provide comprehensive insights into which biophysical indicators contribute most to a participant’s mood scores.
- The generation and analysis of rule-based (IF–THEN) explanations for individual mood scores are presented.

## 2. Materials and Methods

The dataset used in this work was published previously [24]. This dataset was gathered following a one-month study of 14 adult human subjects (with a mean age of 21.6 ± 2.8 years and ten females) before the onset of the COVID-19 pandemic.

### 2.1. Study Summary

Human participants were recruited to the study from the University of California San Diego College Mental Health Program [51]. The study included participants experiencing moderate depression symptoms assessed by using the Patient Health Questionnaire (PHQ-9) scale [52]. Participants with PHQ-9 scores greater than nine were included, with participant scores ranging between 10 and 17. While no structured interview was conducted for this study, suicidal behaviours were screened by using the Columbia Suicide Severity Rating Scale [53]. Any participants on psychotropic medications maintained a stable dose throughout the one-month study, and no participants demonstrated suicidal behaviours during this study. The study protocol was approved by the University of California San Diego institutional review board, UCSD IRB# 180140.

The data were collected through two data-acquisition modes. First, lifestyle and physiological data were collected by using a Samsung Galaxy wristwatch (wearable) that all participants wore throughout the study, except while charging the watch for a few hours once every 2–3 days. Participants also used an application named BrainE on their iOS/Android smartphone [54] to register their daily Ecological Momentary Assessments (EMAs) four times a day for 30 days. During each EMA, participants rated their depression and anxiety on a 7-point Likert scale (with severity increasing from 1 to 7), participated in a 30 s stress assessment and reported their diet (e.g., fatty and sugary food items consumed from a list provided and servings of coffee). Also, neurocognitive and EEG data were collected during assessments in a lab on days 1, 15 and 30 of the one-month study. Participants completed six cognitive assessment games to assess inhibitory control, interference processing, working memory, emotion bias, internal attention and reward processing. Finally, the gathered raw data, which had different sampling frequencies—seconds to minutes for the smartwatch data, hours for the EMA data and days for the neurocognitive data, were reconciled through aggregated or extrapolation to match the sampling frequency of the output variable, i.e., depressed mood scores.

### 2.2. Dataset

The raw dataset contained 48 features (or predictors) for each participant. We removed three speed-based features (such as the cumulative step speed) as they were computed from noisy distance features. Of the remaining 45 features, we chose 43 input features (i.e., inputs to a model), 1 output feature (i.e., the predicted feature) and 1 feature to preserve timing information. The input features included both the smartwatch and neurocognitive-assessment data. Sixteen input features were obtained from the Samsung wearable, and the remaining twenty seven were obtained from the neurocognitive assessments. The wearable and EMA features collected from the smartphone are presented in Table 1. Supplementary Table S1 of [24] describes the remaining features.

**Figure 1:**
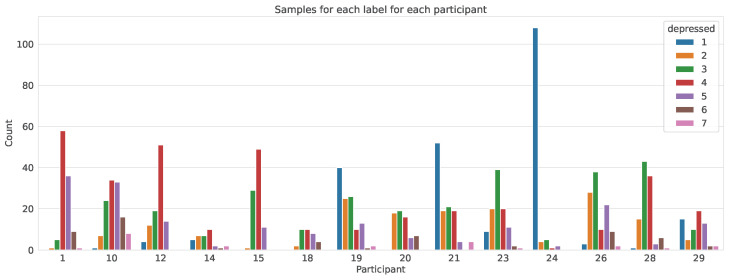
The number of each label/output class per participant. The output class is the mood state captured by the feature *depressed*.

Moreover, the feature *depressed* with a value between 1 and 7 was used as the output feature. The severity of the depressed mood increases from 1 to 7, with 1 indicating feeling not depressed and 7 indicating feeling severely depressed. The *datestamp* feature was used to order the dataset chronologically before any data preprocessing was performed. Table 2 contains sample information for each participant, and Figure 1 shows the output-label distribution for each participant.

As seen from Table 2, nine out of fourteen participants have features where some values are missing. This could be due to device error or participant behaviour (e.g., a participant may forget to wear the smartwatch for a few hours). However, there are no samples where all the feature values/data points are missing. Also, the total number of samples varies between the participants. Participants 14, 18, 21 and 29 have fewer samples, which could have a bearing on the performance of the models [40].

Moreover, we can see from Figure 1 that the label classes (depressed-state values) across participants are not balanced. This is expected as the participants are mild to moderately depressed, and the highest and lowest ends of the depressed mood scale (which correspond to no depression and severe depression, respectively) will be rarely represented. As this is an expected behaviour and we want the model to learn this behaviour, we do not use any methods to balance the dataset prior to training.

Furthermore, we noticed that a few participants (such as Participants 10, 15, 18, 21 and 23) had a few features with constant values, i.e., the same value repeated for each sample. This may make sense for neurocognitive-assessment features (where a participant may perform consistently on the tests) but not for features acquired through the wearable. For instance, a participant would be highly unlikely to have the same nonzero value for features like *exercise calories* or *heart rate* for 30 days. We deal with invalid and missing values in the following data-preprocessing section.

### 2.3. Data Preprocessing

As the dataset contained missing data points and invalid values, we preprocessed the data by using three data-preprocessing methods and built models for each to compare which method suited the dataset. We started with a simple data-preprocessing method and progressively increased the algorithm’s complexity.

For the first method, we used Deletion to ensure that each participant had all 43 features with no missing data points. We began by removing the participants with constant smartwatch feature values. This step eliminated Participants 10, 15, 18, 21, 23 and 24. Then, we removed the samples/rows with any missing data. This step reduced the number of samples for some participants. However, this method was the most straightforward data-preprocessing method we used and provided a good baseline against the more sophisticated data-preprocessing methods discussed next.

For the second method, we used Manual Imputation, which utilised information on the data type (discrete, continuous or neurocognitive) in a feature to impute/fill data. We removed the wearable features (data acquired from the smartwatch) where all values were constant and incorrect. Next, for features with discrete data, the missing values in a feature column were imputed with its most frequent value. In contrast, for features with continuous data, the missing values were imputed by using an iterative method that computes the missing values in each feature by considering it as a function of all other features in a round-robin manner (see Iterative Imputer in Table 3) [55]. Finally, we imputed the missing values in the neurocognitive features with zero, as a zero in an assessment typically implies an empty/void assessment.

For the third method, we employed Automatic Imputation, which automated the imputation stage. We removed the wearable features where all values are constant and incorrect. Next, we handled missing data by choosing a data-imputation method that preserved the original data distribution. Instead of manually choosing an appropriate method, we automated the process and seven different data-filling methods for each feature with missing values. The chosen methods are summarised in Table 3. Finally, we compared the methods by using the distribution of the filled-in feature and the original feature vectors. For this, we used the two-sample Kolmogorov–Smirnov (KS) test, which compares two distributions by finding the maximum difference between the Cumulative Distribution Functions (CDFs) of the two distributions [58]. As different methods were chosen for different features for every participant, we decided against reporting them here to maintain the succinctness of the paper. More information about the approaches discussed in this section can be found in Appendix A.1.1.

### 2.4. Model Development

**Figure 2:**
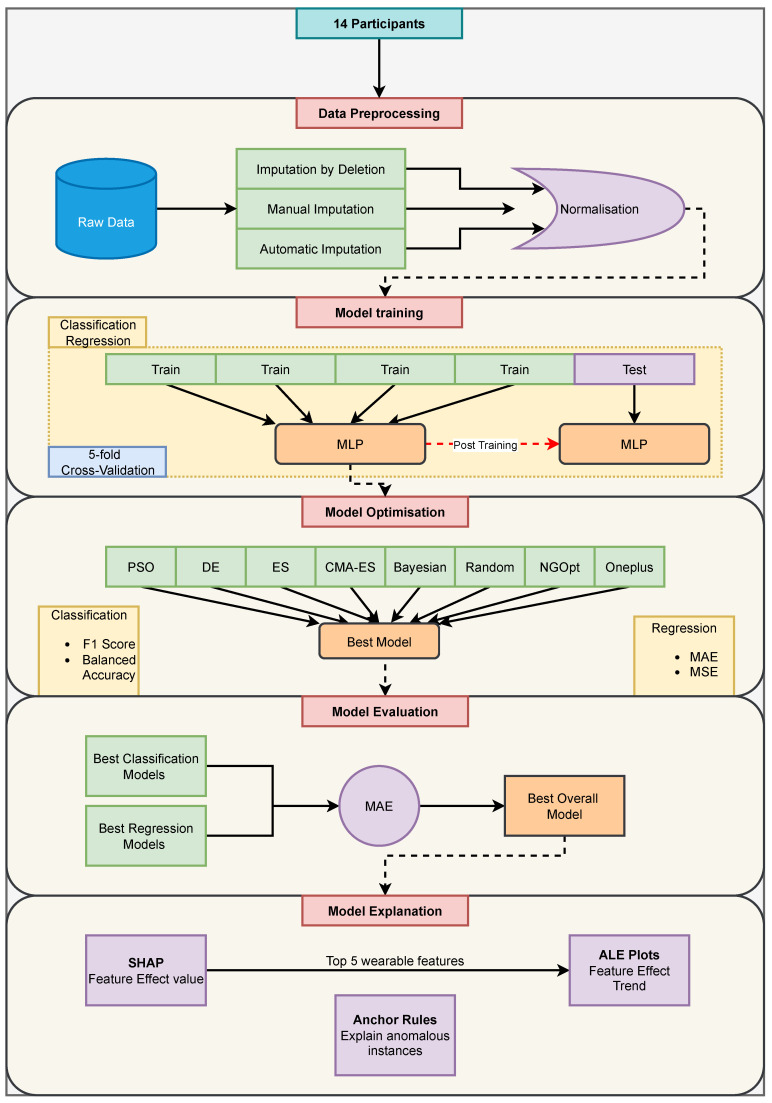
The proposed mood-score-prediction framework for the MLP models discussed in this paper. This framework is repeated for all 14 participants. We begin at the top with data preprocessing. The preprocessed data are then used to train classification- and regression-based MLP models. The best models from the classification and regression training (using 5-fold Cross-Validation) and optimisation are compared to find the best overall models with minimum Mean Absolute Error (MAE). This best overall model is then used to obtain model explanations by using SHAP, ALE plots and Anchor rules.

Since the depression scale is ordinal, i.e., there is an order in the value of the depression/mood score, and it increases from 1 to 7, we can consider the current mood-score-prediction problem as either a regression or classification problem [59]. As a regression problem, the model will be concerned with developing a model that predicts values close to the actual mood scores. On the other hand, a classification model considers the mood scores as seven classes and tries to predict a class based on the input. We used MLP models to build the regression and classification models. Also, we used ten common regression and classification classical ML models to build baseline models against which to compare the performance of MLP models. Moreover, we built the models for the different types of data-imputation schemes (see Section 2.3). The MLP model-development framework is shown in Figure 2.

#### 2.4.1. Base Model

We built a set of base models to act as a baseline for the predictive performance of MLP models on the dataset. We trained ten common classical ML algorithms (eight of which were used in [24]) on the three preprocessed datasets for each participant: Adaboost Regressor, Adaboost Classifier, Elasticnet Regressor, Gradient Boosting Classifier, Gradient Boosting Regressor, Poisson Regressor, Random Forest Classifier, Random Forest Regressor, Support Vector Classifier and Support Vector Regressor. Also, we used a simple grid search (as used in [24]) to tune the hyperparameters of the models. The grid search is a brute-force method that tries all possible combinations of hyperparameters and chooses the combination that provides the best prediction performance.

Furthermore, a Stratified 5-fold Cross-Validation (CV) scheme was used to validate the model performance during and after training. This scheme divides the normalised dataset into five parts, trains a model on the four parts (the training dataset) and tests on the remaining part (the testing dataset). It does so in a round-robin fashion. The division is stratified, meaning each fold contains the same proportion of the different output labels. So, for a 5-fold CV, we built five separate models (with the same architecture) on five training and test datasets. The overall performance was obtained by taking the mean of the training and test performance values over the five sets. Also, the test datasets do not overlap between the folds. This method ensures that the evaluation of the model is free of data-selection bias, which may arise when using a simple train–test split, as the performance depends on the particular split of the train and test set.

For each participant, the model (out of the ten) with the lowest Mean Absolute Error (MAE) after hyperparameter tuning, irrespective of classification or regression, was chosen as the base model. More details on the grid search and the hyperparameters used for each model are provided in Appendix A.3. Note that the base models were only used for performance comparison with the MLP models and were not used for a model-explanation comparison as the explainability of such models in a mood-prediction setting has been explored in [24].

#### 2.4.2. Artificial Neural Networks

Artificial Neural Networks (ANNs) are networks of artificial neurons that attempt to model the behaviour of biological neurons by using mathematical functions composed of linear computations and nonlinear functions called activations, such as *sigmoid*, hyperbolic tangent (*tanh*) and others [40]. Through training, ANNs determine nonlinear relationships between a provided set of inputs and their corresponding outputs. They are often designed as networks of several layers with an input layer, a few hidden layers and an output layer in succession [40]. Many types of ANNs exist, including Convolutional Neural Networks (CNNs) and Recurrent Neural Networks (RNNs), with the most basic type being a Multilayer Perceptron (MLP) network. Once trained over the data, the networks make inferences when exposed to new but statistically similar input data [40]. This ability allows them to perform tasks such as the classification or regression of input data and language translation. MLPs are particularly well suited for tabular data and are used in this work.

#### 2.4.3. MLP Model Architecture

The model architecture differed between the regression and the classification models. As mentioned in the previous section, we used ANNs (MLP) to build the model. While both models had an input layer, a few hidden layers and an output layer, the number of neurons in the output layer differed between the regression and classification model. As a regression model predicts a single continuous output value for each input, all regression models used only one neuron in the output layer with no activation.

On the other hand, the classification models had seven neurons corresponding to the seven classes (mood scores). Outputs from the neurons were normalised (squashed) by using a *softmax* activation. These squashed values (for each neuron) lie between 0 and 1 and represent the probability of an input belonging to that class. The class corresponding to the highest probability value was taken as the output. Model hyperparameters, such as the actual number of layers, the number of neurons in each layer and the activation for each layer, were determined by a hyperparameter-optimisation algorithm described in Section 2.4.5.

#### 2.4.4. MLP Model Training

All models were built and trained in Python by using a loss function and an optimiser. The loss function evaluates the model prediction against the actual output value and produces a numeric value based on how different the prediction and the actual values are. Moreover, the optimiser optimises/modifies the weights/parameters of the ANNs to minimise the loss.

For the classification models, we used a version of the cross-entropy loss (see Equation (1)) called the Sparse Categorical Cross-Entropy. The regression models used either the Mean Squared Error (MSE) or the MAE between the predicted and actual values as the loss function. We used a version of stochastic gradient descent called the Adam [60] optimiser to minimise the loss function $L_{CrossEntropy}$ of all models.

(1) $$L_{CrossEntropy}=\sum_{i=1}^{C}y\cdot \log\hat{y}$$

where *C* is the number of classes in the data, *y* is the expected output and $\hat{y}$ is the predicted output.

The preprocessed dataset was time-sorted based on the timestamps and normalised before being fed into the training models. This normalisation ensures a smoother convergence of the loss function. We used the standard normalisation procedure. It centres the data around zero and gives the dataset a unit standard deviation. In this work, we standard-normalised the preprocessed data by subtracting the feature means (μ) from each feature and dividing the result by the standard deviation (σ) of the feature (see Equation (2)).

(2) $$X=\frac{X-\mu }{\sigma }$$

We used a Stratified 5-fold Cross-Validation (CV) scheme to validate the model performance during and after training, similar to the base-model evaluation. The samples in the normalised folds were then randomised and fed into the MLP models for training, i.e., the MLP models took an input of shape N×F, where *N* is the number of input samples and *F* is the number of features.

Moreover, we followed these steps for all MLP models built for regression and classification, irrespective of the data-imputation method. We trained each model by using batches of train data for 100 epochs, i.e., for 100 iterations of the entire training data (divided into batches). We only saved the best model across the epochs and used the early-stopping strategy, which stops the training before the epochs finish if the model’s performance does not improve for a certain number of epochs. Early stopping helps ensure that the models do not overfit the training data [40]. Figure 2 shows the training framework.

#### 2.4.5. MLP Model Optimisation

It is usually challenging to infer the architecture of an ANN that gives the best possible performance, as multiple model and training parameters often influence the performance of an ANN. Instead of manually choosing and tweaking a few parameters to obtain better performance, as we do with the base models, we used an automated method. We chose multiple Evolutionary Algorithm (EA)-based algorithms and stochastic algorithms to optimise the main model and training parameters (called *hyperparameters* in ML parlance) for a better prediction performance.

We used eight different EA and statistical methods to optimise the number of hidden layers in the model, the number of neurons in the input layer, the activation of the hidden layers and the training batch size. We optimised the number of neurons in the input layer but did not optimise the neurons in each layer as that would increase the number of optimisation variables. Increasing the number of optimisation variables increases the optimisation space, making the optimisation problem more difficult. Instead, we linearly interpolated the neurons in the hidden layers by using the number of neurons in the input layer and the number of neurons in the output layer (which depends on whether the model is classification or regression). The eight EA methods we used and the parameters we modified are mentioned in Table 4. We use N.A wherever the default optimisation parameters were used. Table 5 also contains upper and lower limits for each hyperparameter used during the optimisation.

**Figure 3:**
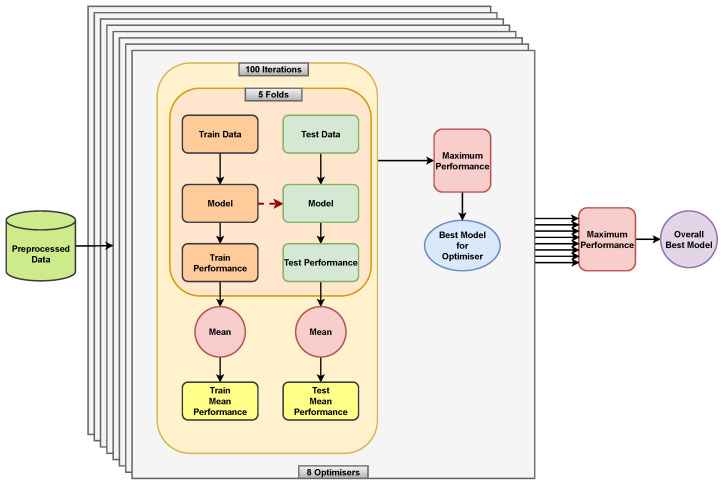
The optimisation procedure shows how the 5-fold Cross-Validation scheme is repeated for 100 iterations for each of the eight optimisers. Different metrics are used to find the best model for the optimiser based on whether the model trained is a classification or a regression model. Similarly, we use different metrics to find the overall best among the eight best models from the optimisers.

Figure 3 shows the optimisation schematic. The hyperparameter optimisation existed as an outer loop to the inner loop of model training (which optimises the model weights). The optimisation repeated over 100 iterations, during which the model was trained by using the 5-fold CV procedure. We averaged the model performance over the five folds and used that as the performance metric to optimise the hyperparameters. We used the metrics F1-score and balanced-accuracy to optimise the classification models and used the MSE and MAE to optimise the regression models. These metrics served as indicators of the model performance and guided the optimisation process towards a set of hyperparameters that provided the best model performance.

At the end of the 100 iterations, we took the best models, i.e., models with the best mean 5-fold performance, from each method and found the best among the eight best (one for each optimisation method) models as well. We used the same metrics to optimise the hyperparameters and find the best models. The performance of these best models was then taken as the best for a particular combination of the optimisation metric, problem type and data-imputation method.

The optimisation procedure was entirely parallelised, and the number of parallel processes was determined by the number of cores in the system used for optimisation and training. We ran the optimisation (and training) in a Docker container containing all the required Python libraries, such as TensorFlow–Keras (for training the models) [66] and Nevergrad (for the optimisation) [65]. Parallelisation significantly reduced the optimisation time, making the procedure scalable to a high number of optimisation iterations.

### 2.5. MLP Model Evaluation and Explanation

We gathered one best model for each combination of problem type (classification or regression), data-preprocessing methodology (3 methods) and metric (2 metrics) used for hyperparameter optimisation (i.e., 12 combinations). As both regression and classification problems used different performance metrics except for the MAE and Mean Absolute Percentage Error (MAPE) (which can be used for both kinds of models), we used the MAE to find the best overall model as it corresponds to the absolute error and not the relative error (like the MAPE).

Hence, for each participant, we collected the best models from the 12 combinations, found the model with the lowest test MAE and used it as the overall best-optimised model. This overall best model was the final model for the participant, and we used this to extract indicators/features that were important as well as to explain how those features affect mood. To this end, we used three post hoc explainability methods from the explainable AI (XAI) literature [36]. We used Shapley Additive Explanations (SHAP) [41], Accumulated Local Effects (ALE) plots [67] and Anchors [68].

SHAP explains a prediction (a single prediction) of a data instance by computing the contribution of each feature to the prediction and is a linear approximation to the Shapley values. They are computed in relation to the average model prediction. Thus, a SHAP value of −0.2 for a feature in a sample, for instance, would mean that the model prediction decreases by 0.2 from the average for a change in that feature. Here, we used SHAP to find the top five important features of each participant. We obtained one SHAP value per data instance per feature, and to compute the global feature importance for a model and a dataset, we took the mean of the absolute SHAP values for all instances in the dataset to obtain the overall SHAP value of a feature. Also, when computing SHAP values, we focused only on features acquired by using wearables and EMAs, as our focus was on finding interventions that could be implemented in a depressed individual’s personal environment, such as at home. Also, as we used a 5-fold Cross-Validation approach, we found the SHAP values for each fold and averaged them.

**Figure A1:**
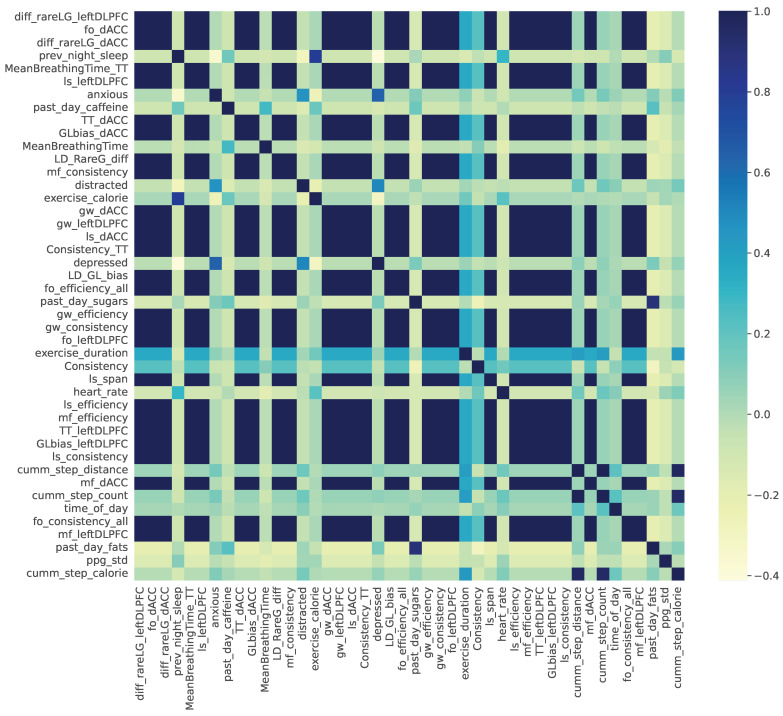
This figure shows the correlation plot for all the input features and output feature used in the models with each other. The bar on the right shows which colour corresponds to which value of correlation. We use Spearman Rank correlation.

Furthermore, ALE plots describe how certain features influence the model prediction, and their value can be interpreted as the main effect of the feature at a certain value compared to the average prediction of the data. ALE works well even when features are correlated and is well suited for our moderately correlated dataset (see plot Figure A1). In this work, we used ALE plots to find how the top-five important features obtained through SHAP influence the model prediction. The plots show how the feature effects on the prediction vary with the value of the feature. This gives us an idea of whether a feature’s increase (or decrease) leads to a corresponding increase (or decrease) in the model prediction compared to the average prediction. As before, we used the test dataset to compute the ALE value for each fold and found the overall ALE value by taking the mean of the ALE values obtained for the five folds.

Finally, Anchors explain a prediction on a data instance of any black-box classification by finding an IF–THEN decision rule that *anchors* the prediction sufficiently. A rule is said to Anchor a prediction if changes in other features do not affect the prediction. Moreover, it includes the notion of coverage, stating which other, possibly unseen instances Anchors apply. We used Anchors to show how specific predictions for classification models could be explained in a rule-based manner. This made Anchors a good candidate to explain anomalous changes in mood. Furthermore, to produce comprehensive rules, we considered all features, including the neurocognitive-assessment features.

Additionally, all post hoc explainability methods take the un-normalised data as the input (which is internally normalised before being fed into the models). Using un-normalised data ensures that the explanations are produced in the actual data range, which makes it easier to interpret. Appendix A.4 contains additional details about the explainability approaches used.

## 3. Results

In this section, we report the results of the best base models and the overall best MLP models, computed as discussed in Section 2.5. As we build personalised models, we report each participant’s results separately. We present the MAE and MAPE and their standard deviations for the best models. Finally, we show the SHAP values, the ALE plots and the Anchor rules for the participant MLP models.

### 3.1. Model Performance

The best MLP model obtained after optimisation is compared to the best base model for each participant. Table 6 shows the average Mean Absolute Error (MAE) and Mean Absolute Percentage Error (MAPE) values of the best base and MLP models on the five-fold Cross-Validation test sets. All models in Table 6 have an MAE of less than or equal to one, which implies that the difference between the actual mood score and the one predicted is, on average, around one. We can also observe that the MAPE values are quite high (around 50%) for some participants even though their MAE values are around one. This is because a high MAPE value can be obtained even if the difference between the actual and predicted mood score is small and the actual mood score is also small. For instance, if the actual mood score is one and the predicted mood score is two, the MAPE value is 100%. However, if the actual mood score is five and the predicted mood score is six, the MAPE value is 20%. Hence, the MAE is a better metric than the MAPE for evaluating these models.

Moreover, the performance (MAE) of the MLP models is better (a lower MAE) than the base models for 10 out of 14 participants, as evidenced by the bold values in Table 6. Although the model hyperparameter search methodology differs between the MLP and the base models, the comparison indicates how powerful the MLP models (a comparatively simple DL method) are at learning meaningful representations for mood scores from digital data. Also, the disparity in model type and performance can be attributed to the differences in the participant datasets used to build the personalised models. Most participant datasets have missing data and high data imbalance (a higher proportion of a specific mood score), and depending on the type of imbalance and amount of good data available, it can make it difficult or easier for certain models to learn associations from them. Overall, MLP models seem to be better able to learn the representations between the input features and the mood score.

Furthermore, Table A1 and Table A2 show hyperparameter combinations and model parameters for the best MLP models reported in Table 6. We find that Deletion and Manual Imputation are the best methods for handling missing data for the participants used in the study. Also, classification models seem to outperform regression models for both the base and MLP models. Of the 14 best MLP models, 9 are classification models and 5 are regression models. Moreover, among the optimisers used to optimise the hyperparameters, Bayesian, DE and PSO are the best methods. There is no visible correlation between the model type, choice of hyperparameters and architecture among the participants, and it seems to be dependent on the participant and the type of model. Appendix A.2 and Appendix A.3 contain more information on the model hyperparameters and architectures used for the best models.

### 3.2. MLP Model Explanation

#### 3.2.1. SHAP Explanation

**Figure 4:**
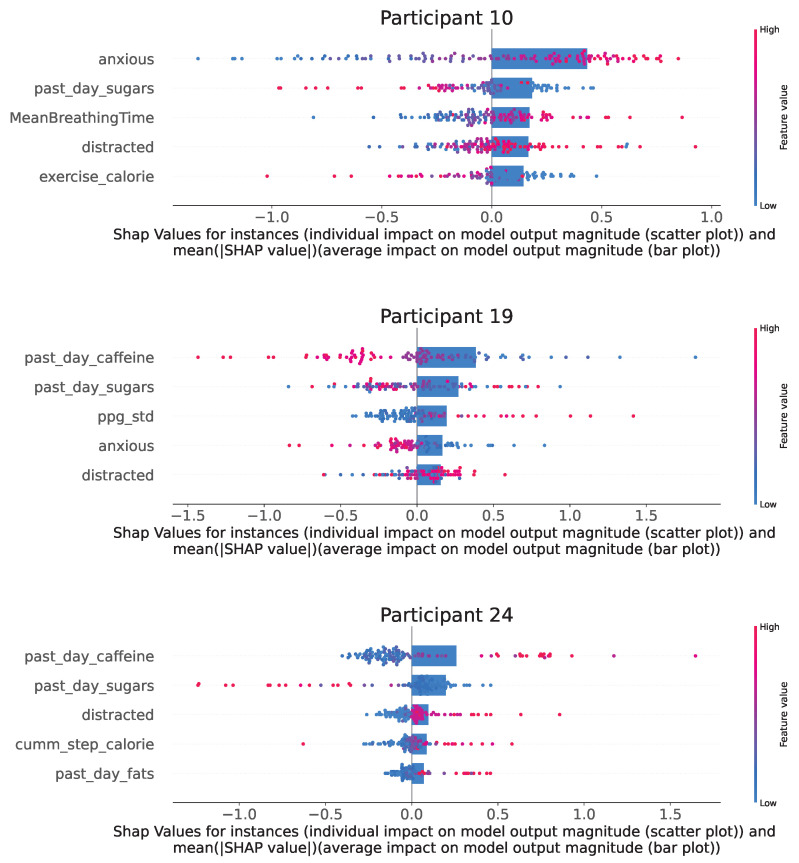
The figure shows the SHAP value effects for the top-5 features in the overall best models for Participants 10, 19 and 24. The scatter plots depict the SHAP values for individual samples, with the colour of the points denoting their magnitude. The bar plots superimposed on top shows the mean of the absolute value of the SHAP values over all data points. The features are arranged based on the magnitude of the average SHAP values. Plots for all participants can be found in Appendix A.5.

Figure 4 shows the five features from the wearable- and EMA-acquired features that affect the model prediction the most based on their SHAP values for three example participants. Plots for all participants can be found in Appendix A.5. SHAP explains a prediction (a single prediction) of a data instance by computing the contribution of each feature to the prediction. We take the mean (average) of the absolute SHAP values for all instances in the dataset and sort them to find the top five features in the figure.

**Figure 5:**
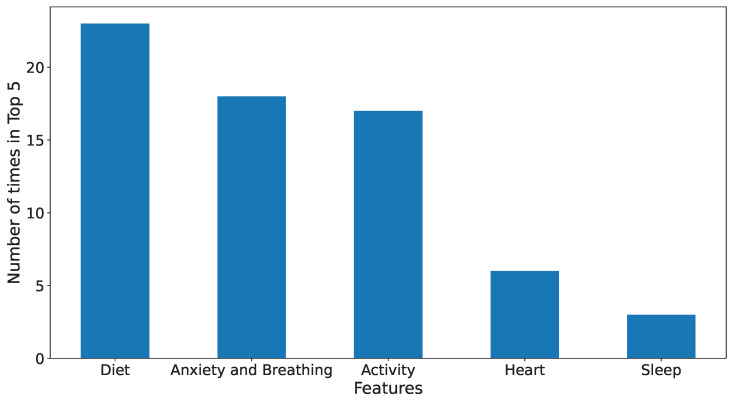
The figure shows the top-5 feature groups in the overall best models for all participants. The features are arranged based on the frequency of their appearance in the top-5 features. The groups contain the following features. Diet: *past-day-fats, past-day-sugars* and *past-day-caffeine*; Anxiety and Breathing: *anxious, distracted, MeanBreathingTime* and *Consistency*; Activity: *cumm-step-count, cumm-step-calorie, cumm-step-distance, exercise-calorie* and *exercise-duration*; Heart: *heart-rate* and *ppg-std*; and Sleep: *prev-night-sleep*.

Figure 5 further shows the overall (population level or for all participants) top feature groups ranked by the number of times they appear in the top-five features, i.e., by their frequency. The figure shows that diet-related features, such as *past-day-sugars*, *past-day-fats* and *past-day-caffeine*, have the highest effect on mood-score prediction. This is followed by anxiety-based features (measured through features like *anxious*, *distracted* and *MeanBreathingTime*). Physical-activity-based features (such as *cumm-step-count and cumm-step-calorie*) have an effect similar to anxiety-based features. Heart- and sleep-based features are the least frequent top-five features and, hence, seem to have the lowest effect on mood scores.

Furthermore, the scatter plot in the figure shows the SHAP value (effect on mood score) for different feature values. There is variability in how certain features affect mood prediction. For instance, for Participant 10, low values for the feature *anxious* (see blue dots for Participant 10 in Figure 4) lead to a decrease in model prediction by one in some instances. Therefore, ensuring lower anxiety for this participant could be a suitable intervention. For instance, an increase in *past-day-caffeine* decreases the mood-score prediction for Participant 19, whereas the opposite is seen for Participant 24.

#### 3.2.2. ALE Plot Explanation

**Figure 6:**
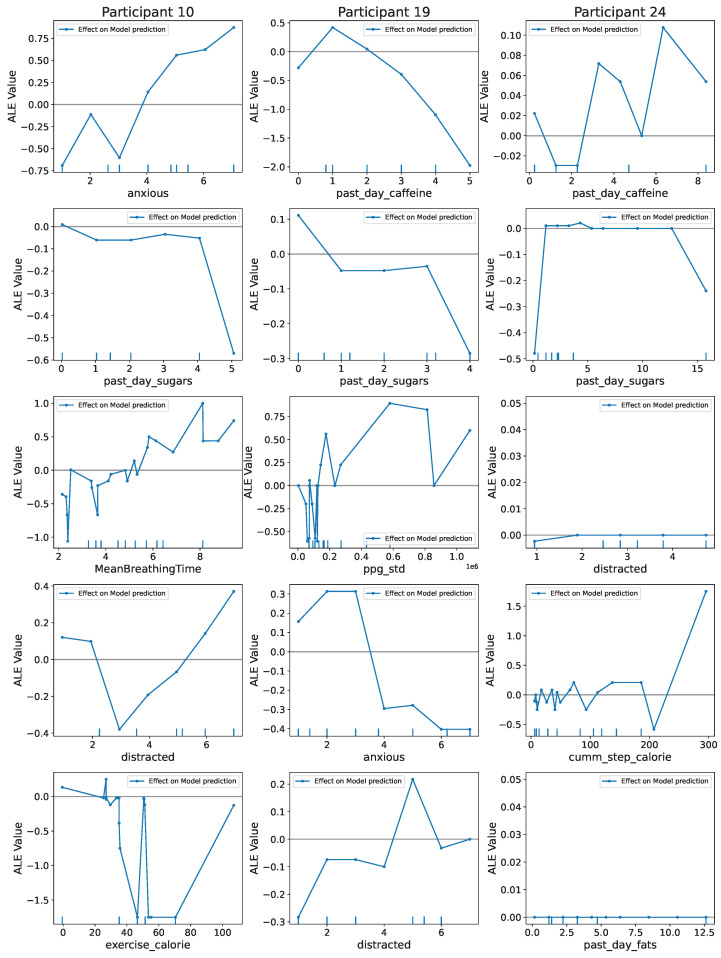
The figure shows the Accumulated Local Effects (ALE) plots for the top-5 features in the overall best models for Participants 10, 19 and 24. The *x*-axis contains the feature values, and the *y*-axis contains the ALE values. The ALE values denote the magnitude of the average effect of a feature value on the model output, i.e., the mood score. Plots for all participants can be found in Appendix A.5.

Although the scatter plots in the SHAP plots show how high and low values of the features affect the model output, they are not good at showing trends. We use ALE plots to see feature trends. Figure 6 shows the ALE plots for the top-five features obtained from the overall best models by using a SHAP value analysis. These plots can help a physician understand how the top predictors influence the model prediction (mood score) with their values. While the plots for some features in some participants are quite simple, others are more complex, denoting a heterogeneity in depressive severity and symptoms. The feature-effect values (the ALE values) for participants will differ from SHAP as ALE plots show only the feature effects separated from any correlation effects between the features (which SHAP does not remove). Also, a positive value (or negative value) on the plot signifies an increase (or decrease) in mood-score prediction. The ALE value in the plot shows the magnitude of such effects.

Taking the example of Participant 10, the ALE value of high *anxious* and *distracted* is high. This implies that a high value of both features leads to an increase, i.e., an overall increase in mood score (or depression). The effect of features is not consistent across participants and may seem counterintuitive. For instance, for Participant 19, an increase in *past-day-caffeine* (the amount of caffeinated items consumed last day) and *past-day-sugars* (the amount of sugary items consumed last day) lead to a decrease in the mood score (less depressed). This may seem counterintuitive as having too many sugary food items is generally unhealthy. In such instances, other explainable methods, such as SHAP and Anchors (as discussed later), could be used to increase trust in the plots. Comparing these results to the SHAP plot (see the scatter plots in Figure 4) for Participant 19, we see that high values of *past-day-caffeine* and *past-day-sugars* decrease the SHAP value or the model prediction of mood score. As the SHAP explanation aligns with the findings of the ALE plots, it increases our trust in the ALE findings.

Figure 6 also shows that for some participants, such as Participant 24, the feature effect of *distracted* is nearly zero. This does not necessarily mean the feature is unimportant or has no trends. It may happen if one half of the dataset exerts a positive effect and the other half a negative effect, one half cancelling the effect of the other half during averaging. Not much helpful information can be gathered from the plots in such instances. Nevertheless, ALE plots are one of the best to visualise any feature’s true-effect trends in the presence of interactions/correlation between features.

#### 3.2.3. Anchors Explanation

Finally, we show how Anchors can explain unusual mood scores by finding the best decision rules (predicated on the features) that apply to a prediction. For instance, a participant’s mood score may increase by three points (e.g., from 3 to 6), signifying a sudden increase in depressive mood. We tag such instances as anomalous instances and try to explain the prediction at and before the increase. By comparing the IF–THEN-based rules obtained from Anchors before and after the anomaly, we can surmise what may have changed in the observed features to elicit such a mood change.

The Anchors procedure takes in a sample corresponding to a mood-score prediction, perturbs the sample to create artificial samples in the neighbourhood of the original sample and finds the region (rules) in the perturbed neighbourhood where the decision rules do not change the prediction. Since this method can only be used for classification models, we use it on participants where the best model is a classification model. Also, we only use it for instances where the prediction was correct to ensure that the rules have fidelity.

Also, Anchors allow the user to specify the maximum number of features to use when finding the rules. This allows the user the choice between concise or comprehensive rules. To show how Anchor rules can be constructed and interpreted, we use anomalous instances from Participants 1, 10 and 24. We begin with the case where the mood score for Participant 1 increases from three to five within 19 h and use a maximum of five features to construct the rules:

(3) $$\begin{array}{c} IF\;[(past-day-fats\leq 11.40\;items)\wedge (GLbias-dACC>0.00)]\;THEN;\\ Depressed=3\\ Precision:1\\ Coverage:0.20\\ \;IF\;[(past-day-sugars>25.00\;items)\wedge (distracted>5.00)\wedge (cumm-step-calorie\leq 285.62)]\\ THEN;Depressed=5\\ Precision:0.95\\ Coverage:0.03 \end{array}$$

Equation (3) shows the conditions/rules on the features needed to ensure that the mood-score prediction (*Depressed* in the equation) changes from three to five, where GLbias−dACC is the neural activity in the dACC brain region corresponding to bias for frequent gains (see the appendices section in [24]). The first set of conditions pertains to the case when the mood score is three, and the second set is when the score is five. Furthermore, the precision and coverage values in the Anchors in Equation (3) say that the Anchors applied to 20% (3% (in the second set of conditions)) of the perturbation-space instances and 100% (95%) of those instances conformed to the rule, i.e., had a prediction of three (five). Higher precision and coverage imply greater fidelity of Anchor rules to the model behaviour.

If we compare the Anchors, we observe that less physical activity, being more distracted and consuming significant amounts of sugary food items (more than 25) are associated with a high mood score/depressive mood. Also, it appears that having fewer fatty food items helps keep the mood score (depression) moderately low (at three). Next, we look at the instance when the mood score for Participant 24 increases from 0 to 4 in 4 h and use two as the maximum number of features to use when constructing the rules (see Equation (4)):

(4) $$\begin{array}{c} IF\;[(distracted\leq 2.84)\wedge (past-day-fats\leq 3.28\;items)]\;THEN;\\ Depressed=0\\ Precision:0.98\\ Coverage:0.38\\ \;IF\;[(past-day-caffeine>5.32\;servings)\wedge (MeanBreathingTime\leq 3.38)]\;THEN;\\ Depressed=4\\ Precision:0.58\\ Coverage:0.01 \end{array}$$

Equation (4) shows that having more than five servings of coffee and breathing faster than 3.38 s per breath contributes to a sudden increase in the mood score (increase from 0 to 4 in 4 h), whereas being less distracted and consuming less fatty items is associated with a very low mood score of 0. Decreasing coffee and fat intake and breathing exercises could help this participant keep sudden variations in their mood scores in check. Finally, we look at the instance when the mood score for Participant 10 decreases from four to two in 11 h. We present the Anchors rules with better precision and coverage between five and two maximum features (Equation (5)):

(5) $$\begin{array}{c} IF\;[(anxious>4.04)\wedge (distracted\leq 2.94)]\;THEN;\\ Depressed=4\\ Precision:0.80\\ Coverage:0.08\\ \;IF\;[(fo-leftDLPFC>0.00)\wedge (anxious\leq 2.03)\wedge (heart-rate\leq 88.16)]\;THEN;\\ Depressed=2\\ Precision:1\\ Coverage:0.08 \end{array}$$

We can gather from Equation (5), where fo−leftDLPFC is the neural activity in the left DLPFC brain region evoked by the Face Off emotion bias task, that having low anxiety and a low heart rate is associated with a low mood score of two, whereas having an anxiety level of more than four contributes to a higher mood score. Clearly, for Participant 10, keeping anxiety levels low is key to managing depression.

Although Anchor rules provide good human-readable explanations to instances, it may be difficult to find rules if the maximum number of features chosen to construct the rules is insufficient. The second rule in Equation (4) is an excellent example of this situation with a precision of only 58%. Increasing the maximum feature size could help increase the precision and our confidence in the rule. Also, finding more Anchors around the anomaly and future anomalies should provide a good idea of what feature variations lead to a change in the mood score. A similar strategy can also be adopted for other participants to explain normal instances. We could also extend the use of Anchors to describe a period of unusual mood scores by explaining all instances within the period and finding the common rules within the instances. A similar analysis can be obtained for participants not presented here.

## 4. Discussion and Conclusions

Depression affects a large population worldwide and has a substantial global healthcare burden [2,69]. With the amount of technology around us, we are generating significant amounts of data. The recent literature has focussed on using data-driven methodologies to create predictive models for depression [14,15,16,17,18,19,21,25,26,27,28,29,30,31]. With the variability seen among depressed people [33,34], personalised predictive models have been suggested in recent years [11].

Hence, we proposed a novel explainable framework to utilise multimodal data to build personalised and explainable Deep Learning (DL) models for people experiencing depression. To illustrate the framework, we used a dataset with 14 mild to moderately depressed participants from a previously published work [24]. The dataset, collected over one month, contained activity data from a smartwatch, diet and mood-assessment reports from Ecological Momentary Assessments (EMAs) and neurocognitive data from in-person sessions. We preprocessed the raw data through multiple data-imputation schemes and trained both classification- and regression-based MLP (Multilayer Perceptron) models to produce predictions of mood scores—a discrete score based on the severity of their depressive symptoms.

The models are optimised through eight Evolutionary and Statistical optimisation algorithms to find the hyperparameters that offer the best model performance evaluated by using a five-fold Cross-Validation model training routine to obtain a robust estimate of the model performance. We compared this performance against ten classical ML-based baseline models and showed how the MLP models outperformed the baseline models. The best-performing MLP models were further analysed by using SHAP (Shapley Additive Explanations) [41] and ALE (Accumulated Local Effects) [67] plots to extract the top features/indicators that influenced the model and reveal the associations between the top feature indicators and depression. Moreover, we demonstrated how rule-based explanations predicated on features could be generated from the models by using Anchors [68]. Such explanations can potentially guide clinical or self-management interventions for depression.

Our work differs from previous research on explainable depression modelling through mood-score prediction in many ways. Works like [35,70] perform an analysis on cross-sectional datasets, whereas we use a longitudinal dataset. Moreover, most studies, like [21,25], employ simple ML to build predictive models for depression, and studies that employ DL, like [26,28], do not employ a parallel, multiple Evolutionary Algorithm-based optimisation scheme to optimise the model hyperparameters. Furthermore, most studies like [46,47,48,49] use explainability to develop population-level explanations of various mental health disorders, while our work produces personalised insights.

The work by Shah et al. [24] shares the most similarity with our work and develops personalised mood-prediction models on the same dataset and uses methods from the explainable AI literature [36]. However, it uses it primarily to extract features (using SHAP) that have the most influence on the model’s prediction of the mood/depression score. We extend their work by using Accumulated Local Effects (ALE) plots to show how changes in the value of such features influence the model’s prediction of mood scores. We pipeline SHAP and ALE to show how the top wearable- and EMA-based features affect mood scores. We focus on these features as their trends allow one to suggest interventions based on lifestyle, such as diet and activity, as they can be monitored comfortably in real time in a person’s usual environment. We further generate rule-based (IF–THEN) explanations for instances showing sudden changes in the mood score (increase or decrease in depression) using Anchors. These rule-based explanations include bounds on features, which can be used to quantify interventions by using those features.

In general, we found that the MLP models were better able to learn the representations between the input features and the mood score. Although the model-hyperparameter search methodology differed between the MLP and the base models, the results indicate how powerful MLP models (a comparatively simple DL method) are at learning meaningful representations for mood scores from digital data. Moreover, our results on the top-five features for individuals slightly differed from that in [24] due to the differences in model types, data preprocessing and feature design. Interestingly, the results for the population-level top-five features were similar to that in [24], with diet- and anxiety-related features being the most frequent top-five features.

We also found that SHAP and ALE plots had the potential to help clinicians find the most influential features/indicators for intervention and how their values influence the mood score. Moreover, human-readable rules from Anchors could help clinicians obtain a quantitative estimate of feature limits (range) for individual predictions of mood scores in depressed individuals. By observing the feature ranges in the rules over time, a clinician could advise interventions focussed on certain activities and food items. The numerical bounds in the rules should help determine the limits for such interventions.

The overall framework presented in this work can be extended to other kinds of modelling approaches, data types and optimisation schemes; however, the results presented in this study are limited by the dataset (quality and quantity) used and some of the shortcomings of the explainability approaches used. The dataset for some participants has missing and invalid data. Even though the data-imputation schemes handled both issues, a complete dataset for those participants could have yielded more performant models and better explanations.

Moreover, one of the pitfalls when analysing models by using *explainable* methods is the need for clarity between causation and association. All explainability methods discussed here only provide information on the association, not causation. For instance, if an increase in the feature *anxious* is seen to increase the mood score in an ALE plot, we cannot say that being more anxious causes a participant to be more depressed. It could be the case that an increase in depression causes an increase in anxiety for that participant. Therefore, all we can say is that an increase in anxiety is associated with an increase in depression.

Furthermore, explainability methods are model-based, and the explanations produced are explanations for the model and not the underlying data distribution. This implies that if the model is poor, the explanations produced by using the model will not be reliable either. Thus, for the two participants (Participant 19 and Participant 21), where our models had a high MAPE value, the explanations (important features and feature trends) may be unreliable. Also, there may be instances where the explanations obtained from one of the three methods discussed in this work may seem counterintuitive. In such instances, we propose validating the results through the remaining two explainability methods.

Personalised models for depression by using wearable and other relevant data provide an opportunity for personalised treatment approaches as long as data of good quality and quantity are available and the pitfalls associated with using model-explainability methods are understood. Accurate, personalised models and the explanations generated from them can help build associations between individual activities and depression severity, assisting medical professionals and patients in managing depression through targeted interventions. This work presents a framework to achieve this. In the future, a combination of cross-sectional and longitudinal methodologies could solve the data quantity problem. Also, work on incorporating other modalities of data, such as speech and facial emotions, and different kinds of models, such as timed DL models, could improve the predictive models further.

## Figures and Tables

**Table 1:** Summary of features acquired using EMA and smartwatch.

#	Feature	Description
1	distracted	EMA-based 1–7 ratings of “How distracted do you feel right now?” acquired four times per day alongside the depressed-mood ratings
2	anxious	EMA-based 1–7 ratings of “How relaxed versus anxious do you feel right now?” acquired four times per day alongside the depressed-mood ratings
3	MeanBreathingTime	Mean breathing time of the 30 s active stress assessment acquired four times 4× per day alongside the depressed-mood ratings
4	Consistency	Consistency of breathing in the 30 s active stress assessment acquired 4× per day alongside the depressed-mood ratings
5	past-day-fats	Total fatty items consumed in the 24 h prior to each depressed-mood rating
6	past-day-sugars	Total sugary items consumed in the 24 h prior to each depressed-mood rating
7	past-day-caffeine	Total cups of caffeine consumed in the 24 h prior to each depressed-mood rating
8	heart rate	Smartwatch-based heart rate as the mean heart rate in the ±30 min window around each depressed mood EMA
9	ppg-std	Heart Rate Variability from the Tizen Photoplethysmography data as the standard deviation within the ±15 min window around each depressed-mood EMA
10	cumm-step-count	Cumulative step count taken as the mean value from the past 12 h of each depressed-mood rating
11	cumm-step-calories	Cumulative step calories burnt taken as the mean value from the past 12 h of each depressed-mood rating
12	cumm-step-distance	Cumulative step distance taken as the mean value from the past 12 h of each depressed-mood rating
13	cumm-exercise-calories	Cumulative exercise calories burnt taken as the mean value from the past 24 h of each depressed-mood rating
14	cumm-exercise-duration	Cumulative exercise duration taken as the mean value from the past 24 h of each depressed-mood rating
15	prev-night-sleep	Number of hours of sleep the previous night of each depressed-mood rating
16	time_of_day	Time of the day when a particular depressed-mood rating was taken: (6:00, 10:00], (10:00, 14:00], (14:00, 18:00] and (18:00, 23:59]

**Table 2:** Summary of samples for each participant. The Missing Values column shows the number of missing data points out of all the data points for the participant, which is 43 (number of features) times the total number of samples for that participant.

Participant	Total Samples	Missing Values (Out of 43 × Total Samples)	Features with Missing Values
1	110	28	1
10	123	108	27
12	100	116	31
14	34	1	1
15	90	0	0
18	34	0	0
19	117	18	1
20	66	11	1
21	119	0	0
23	102	0	0
24	120	21	1
26	112	10	4
28	105	0	0
29	66	9	1

**Table 3:** Summary of methods used for handling missing data in a feature column.

Method	Description
Mean	Fill missing data with the mean of the available data
Median	Fill missing data with the median of the available data
Forward fill	Fill missing data by continuing the last available data
Backward fill	Fill missing data by continuing the next available data
Linear interpolation	Fill missing data through linear interpolation by using the data points before and after the missing data
Iterative Imputation [56]	A strategy for imputing missing values by modelling each feature with missing values as a function of other features in a round-robin fashion. This method only uses samples with no missing data as the input (in case multiple features in a data point have missing values)
KNN Imputation [57]	Each sample’s missing values are imputed by using the mean value from some nearest neighbours in the training set. Two samples are close if the features that neither are missing are close.

**Table 4:** Summary of optimisation algorithms/methods used in hyperparameter optimisation.

Algorithm	Summary	Parameters
PSO [61]	Particle Swarm Optimisation of the loss on hyperparameters based on a set of particles (candidate solutions) with their inertia	Population size: 10
DE [62]	Differential Evolution-based optimisation. It uses differences between points in the population (candidate solution) for doing mutations in fruitful directions	Population size: 10
ES [63]	Evolution Strategy-based optimisation of hyperparameters is based on the ideas of evolution	Population size: 10
CMA ES [63]	Covariance Matrix Adaptation Evolutionary Strategy is a variation in ES where the covariance matrix of the distribution is incrementally updated to increase the likelihood of previously successful search steps	Population size: 10
Random	Random search of the hyperparameter space	N.A
Bayesian [64]	Bayesian Optimisation of the loss on hyperparameters. Optimisation of search space depends on the initialisation type	Initialisation: Latin Hypercube Sampling
NgOpt [65]	Nevergrad Library’s own optimiser	N.A
Oneplusone [65]	1 + 1 optimisation of the loss on the hyperparameter space	N.A

**Table 5:** Search values (or limits) for parameters during hyperparameters optimisation.

Hyperparameter	Lower Limit	Upper Limit	Values
Number of layers	2	4	N.A
Neurons in input layer	30	60	N.A
Activation	N.A	N.A	tanh, relu, elu, linear
Batch size	N.A	N.A	4, 6, 8

**Table 6:** Table containing the metrics for the best base and MLP models for each participant. The values in bold indicate lower values. (MAE: Mean Absolute Error; MAE STD: Mean Absolute Error standard deviation; MAPE: Mean Absolute Percentage Error in %; MAPE: Mean Absolute Percentage Error standard deviation in %; P-1 to P-29 refer to Participants 1 to 29, respectively).

Subject ID	Model	MAE		MAPE		Subject ID	Model	MAE		MAPE	
		Mean	STD	Mean	STD			Mean	STD	Mean	STD
P-1	Support Vector Classifier	0.336	0.145	7.971	2.920	P-10	Random Forest Classifier	**0.697**	0.113	**18.911**	5.051
	MLP Classifier	**0.295**	**0.151**	**6.155**	**2.729**		MLP Classifier	0.715	**0.089**	19.839	**4.426**
P-12	Support Vector Classifier	**0.575**	0.077	27.900	7.483	P-14	Support Vector Classifier	0.904	**0.146**	46.644	20.866
	MLP Regressor	0.582	**0.063**	**22.260**	**3.107**		MLP Regressor	**0.829**	0.327	**36.152**	**17.918**
P-15	Gradient Boosting Regressor	**0.467**	0.115	14.296	5.028	P-18	Random Forest Classifier	0.823	**0.185**	22.047	**8.075**
	MLP Regressor	0.494	**0.050**	**13.114**	**1.583**		MLP Classifier	**0.638**	0.472	**18.857**	18.541
P-19	Gradient Boosting Regressor	1.072	0.171	60.931	19.505	P-20	Random Forest Classifier	0.636	**0.181**	19.757	6.854
	MLP Classifier	**0.989**	**0.096**	**53.204**	**12.124**		MLP Classifier	**0.455**	0.249	**13.515**	**6.165**
P-21	Random Forest Classifier	1.152	0.298	**41.876**	16.714	P-23	Gradient Boosting Regressor	**0.883**	**0.307**	38.242	**6.940**
	MLP Regressor	**1.103**	**0.216**	53.113	**11.710**		MLP Classifier	0.936	0.550	**38.075**	17.184
P-24	Support Vector Classifier	0.158	**0.074**	6.736	5.124	P-26	Gradient Boosting Regressor	1.098	0.213	33.008	9.015
	MLP Classifier	**0.125**	0.088	**5.347**	**4.933**		MLP Regressor	**1.013**	**0.149**	**32.206**	**8.699**
P-28	Random Forest Classifier	0.609	0.173	19.710	6.624	P-29	Adaboost Regressor	1.188	0.310	60.830	**12.391**
	MLP Classifier	**0.562**	**0.163**	**17.565**	**6.398**		MLP Classifier	**1.03**	**0.253**	**47.369**	18.945

**Figure A2:**
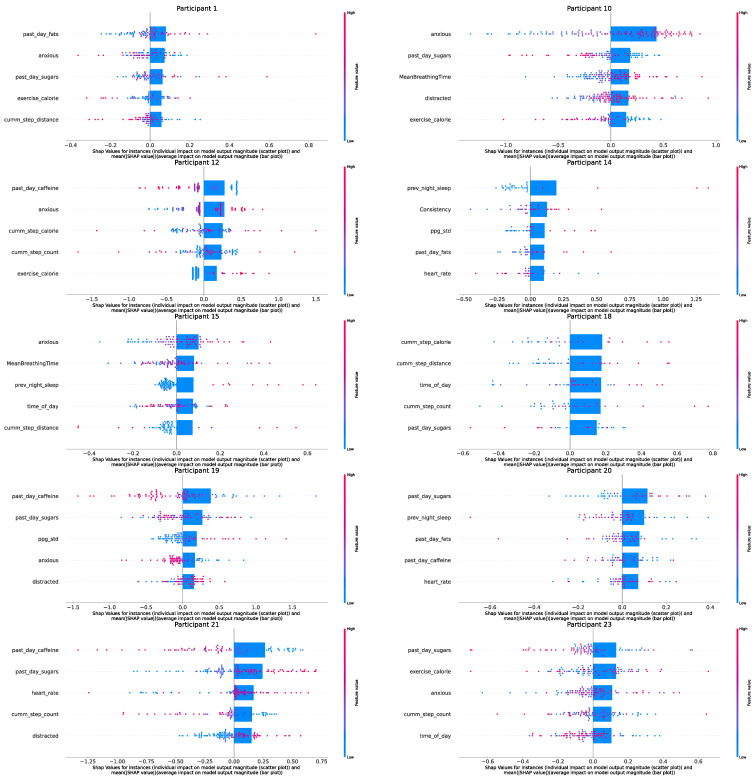

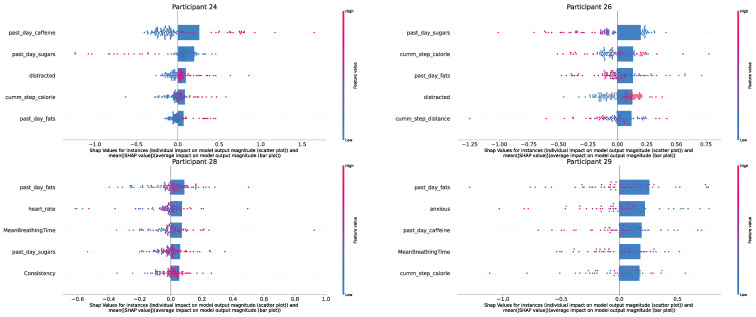
This figure shows the SHAP value effects for the top-5 features in the overall best models for all participants. The scatter plots depict the SHAP values for individual samples, with the colour of the points denoting their magnitude. The bar plots superimposed on top show the mean of the absolute value of the SHAP values over all data points. Figure continues on next page.

**Figure A3:**
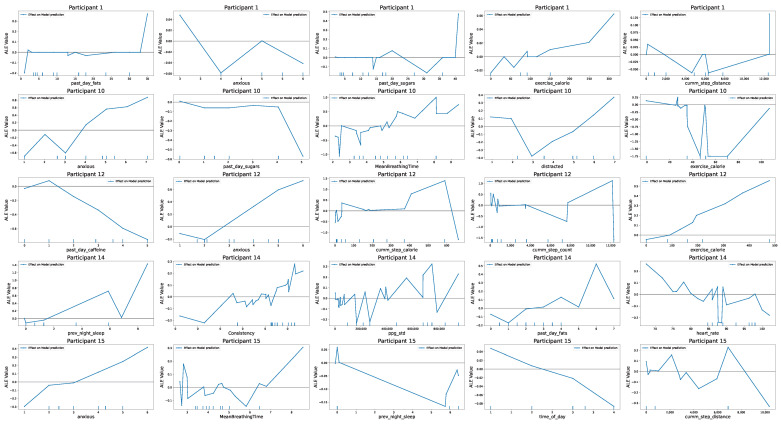

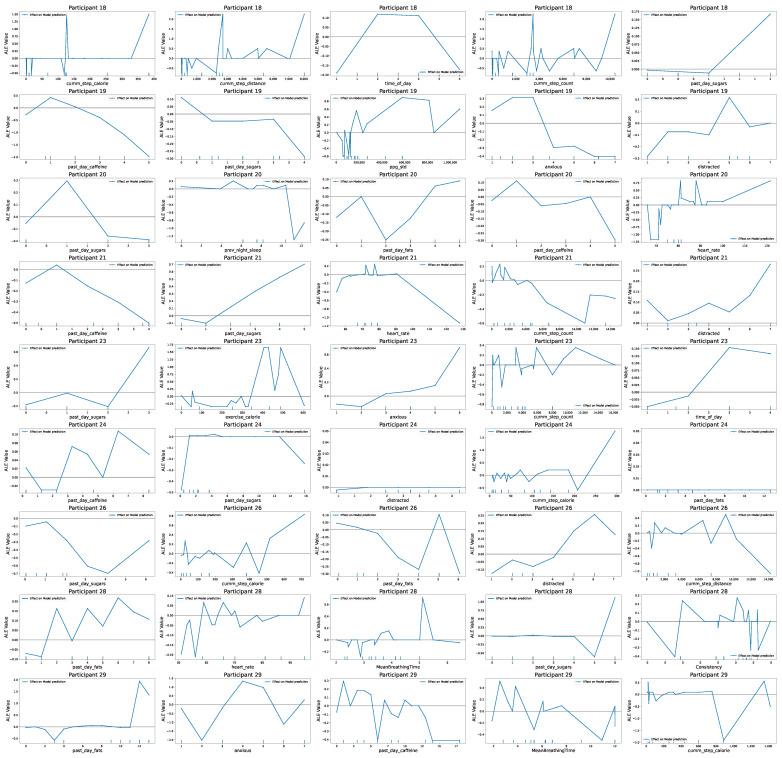
This figure shows the Accumulated Local Effects (ALE) plots for the top-5 features in the overall best models for all participants. The *x*-axis contains the feature values, and the *y*-axis contains the ALE values. The ALE values denote the magnitude of the average effect of a feature value on the model output, i.e., the mood score. Figure continues on next page.

## Data Availability

The participant data used in the study cannot be divulged due to ethical reasons. The analytics data are available upon request from the corresponding author.

## Appendix A

### Appendix A.1. Data Information

#### Appendix A.1.1. Data Preprocessing

As different data-preprocessing methods have advantages and disadvantages, we try three data-preprocessing methods and build models for each to compare which method suits the dataset.

For the first method, we use Deletion as follows:

- Identify participants for whom we have constant feature values for features acquired from the smartwatch—call it set *C*. Remove participants in *C*.
- Identify (from remaining participants) those participants for whom we have missing data points—call it set *M*. For participants in *M*, remove the samples containing the missing data points.

For the second method, we use a Manual Imputation technique by using a set of differentiated data-preprocessing steps as follows:

- Identify participants for whom we have constant feature values for features acquired from the smartwatch—call it set *C*. Remove the features with constant values for participants in *C*
- Identify participants for whom we have missing data points—call it set *M*. For participants in *M*, identify the features with missing data points—call it set *F*.
- Identify if the features in set *F* contain discrete, continuous or neurocognitive values.
- For discrete features, the missing values are filled with the most frequent value.
- For continuous features, the missing values are imputed by using an Iterative Imputer (see Table 3).
- For neurocognitive features, we fill in the missing values with zero.

For the third method, we employ Automatic Imputation by using a set of sophisticated data-preprocessing steps as follows:

- Identify participants for whom we have constant feature values for features acquired from the smartwatch—call it set *C*. Remove the features with constant values for participants in *C*
- Identify participants for whom we have missing data points—call it set *M*. For participants in *M*, identify the features with missing data points—call it set *F*.
- For each feature in *F*, fill in the missing values with multiple data-imputation methods, one at a time.
- Choose the data-imputation method that gives a data distribution that best matches the data distribution before any preprocessing.

In Automatic Imputation, we compare the methods by using the distribution of the filled-in feature and the original feature vectors. For this, we use the two-sample Kolmogorov–Smirnov (KS) test, which compares two distributions by finding the maximum difference between the Cumulative Distribution Functions (CDFs) of the two distributions [58]. In general, the KS test is used to compare the underlying continuous distributions F(x) and G(x) of two independent samples. This statistical test uses the null hypothesis that the two distributions are identical, i.e., F(x) = G(x) for all x. The alternative hypothesis is that they are not identical. The test produces a *statistic* and a *p*-value [71]. The *statistic* obtained from the test is the maximum absolute difference between the empirical distribution functions of the samples.

We choose a confidence level of 95%; i.e., we reject the null hypothesis in favour of the alternative if the *p*-value is less than 0.05. The higher the *p*-value, the more probable the fact that the two distributions (i.e., before and after data filling) are similar. Thus, we choose a method with the highest *p*-value and lowest *statistic*. As different methods are chosen for different features for every participant, we decided against reporting them here to maintain the succinctness of the paper.

### Appendix A.2. MLP Model Hyperparameters

Table A1 shows the combination of the data-preprocessing methodology, the problem type and the metric used to optimise the model hyperparameters. The optimiser for which we obtain the overall best model is also provided. Table A2 shows the model parameters for the overall best models. It presents the number of neurons in the DNN, the activation used in the layers and the batch size used during the training of the models. The table shows that most models have at least three layers (two hidden layers and one output layer) and use either a ReLU or a linear activation. However, for Participants 15, 18 and 28, the DNN only has one hidden layer. Furthermore, a batch size of six or four samples works best with most participants.

**Table A1:** Combinations yielding the overall best models. Metric refers to the metric used to optimise the model hyperparameters, and Optimiser refers to the best optimiser (used for hyperparameter optimisation) corresponding to the overall best model. (BA: balanced-accuracy, F1: F1-score, MAE: Mean Absolute Error and MAPE: Mean Absolute Percentage Error).

Participant	Data Preprocessing	Problem Type	Optimiser	Metric
1	Deletion	Classification	DE	BA
10	Manual Imputation	Classification	Bayesian	F1
12	Deletion	Regression	CMA-DE	MAE
14	Deletion	Regression	PSO	MAE
15	Manual Imputation	Regression	PSO	MAE
18	Manual Imputation	Classification	Bayesian	BA
19	Deletion	Classification	Bayesian	BA
20	Deletion	Classification	DE	BA
21	Manual Imputation	Regression	ES	MAE
23	Manual Imputation	Classification	DE	BA
24	Automatic Imputation	Classification	Bayesian	F1
26	Manual Imputation	Regression	PSO	MAE
28	Deletion	Classification	PSO	F1
29	Deletion	Classification	Bayesian	F1

**Table A2:** Parameters of the overall best models. Neurons column contains the neurons for each layer (hidden + output layers) in the DNN. ReLU: Rectified Linear Unit, ELU: Exponential Linear Unit, tanh: hyperbolic tangent and Linear: no nonlinear activation.

Participant	Neurons	Activation	Batch Size
1	{38, 7}	ReLU	6
10	{60, 33, 7}	Linear	8
12	{46, 31, 16, 1}	Linear	4
14	{30, 20, 10, 1}	ReLU	4
15	{48, 1}	ReLU	6
18	{52, 7}	Linear	6
19	{35, 21, 7}	Linear	6
20	{45, 32, 19, 7}	ELU	6
21	{50, 25, 1}	Tanh	4
23	{36, 21, 7}	ReLU	8
24	{51, 29, 7}	Linear	6
26	{46, 31, 16, 1}	ReLU	4
28	{60, 7}	ReLU	8
29	{51, 36, 21, 7}	ReLU	4

### Appendix A.3. Base-Model Hyperparameters

The grid search is implemented in Python by using a combination of Python libraries. The Scikit-learn [72,73] library is used for the modelling, and further information on the model parameters reported in the following subsections can be found on their API page. The following sections contain information about the grid search parameters and the results of the search.

#### Appendix A.3.1. Adaboost Classifier

The grid search parameter sets are as follows. Estimators ∈ {50, 100, 200}, Learning Rate ∈ {0.5, 1, 2, 10}, and Algorithm ∈ {‘SAMME’, ‘SAMME.R’}.

**Table A3:** Parameters of the Adaboost Classifier for the best model after grid search.

Participant	Data Preprocessing	Estimators	Learning Rate	Algorithm
1	Impute	200	1	SAMME.R
10	Preprocessed	50	0.5	SAMME
12	Deletion	100	0.5	SAMME.R
14	Deletion	200	2	SAMME.R
15	Deletion	200	0.5	SAMME
18	Impute	200	10	SAMME
19	Deletion	50	0.5	SAMME
20	Deletion	200	1	SAMME
21	Preprocessed	50	2	SAMME
23	Impute	200	10	SAMME.R
24	Deletion	50	1	SAMME.R
26	Impute	100	0.5	SAMME
28	Deletion	50	0.5	SAMME
29	Deletion	100	1	SAMME

#### Appendix A.3.2. Adaboost Regressor

The grid search parameter sets are as follows. Estimators ∈ {50, 100, 200}, Learning Rate ∈ {0.5, 1, 2, 10}, and Loss ∈ {linear, square, exponential}.

**Table A4:** Parameters of the Adaboost Regressor for the best model after grid search.

Participant	Data Preprocessing	Estimators	Learning Rate	Loss
1	Deletion	50	2	Exponential
10	Preprocessed	50	0.5	Exponential
12	Deletion	100	1	Square
14	Deletion	50	0.5	Exponential
15	Deletion	200	2	Linear
18	Preprocessed	50	2	Linear
19	Impute	50	0.5	Linear
20	Deletion	200	2	Linear
21	Deletion	50	1	Square
23	Preprocessed	200	10	Exponential
24	Deletion	50	1	Square
26	Preprocessed	100	10	Exponential
28	Impute	100	10	Exponential
29	Deletion	50	1	Square

#### Appendix A.3.3. Elasticnet Regressor

The grid search parameter sets are as follows. Alpha ∈ {0.5, 1, 2, 10}, L1-Ratio ∈ {0, 0.5, 1}, and Selection ∈ {random, cyclic}.

**Table A5:** Parameters of the Elasticnet Regressor for the best model after grid search.

Participant	Data Preprocessing	Alpha	L1-Ratio	Selection
1	Impute	2	0	Cyclic
10	Impute	0.5	0	Cyclic
12	Deletion	0.5	0	Random
14	Deletion	0.5	0.5	Random
15	Preprocessed	10	0	Cyclic
18	Deletion	1	0	Random
19	Deletion	1	0	Random
20	Deletion	2	0	Cyclic
21	Impute	10	0	Cyclic
23	Deletion	0.5	0.5	Cyclic
24	Deletion	10	0	Cyclic
26	Impute	0.5	0	Cyclic
28	Deletion	1	0	Random
29	Deletion	10	0	Cyclic

#### Appendix A.3.4. Gradient Boosting Classifier

The grid search parameter sets are as follows. Loss ∈ {log-loss}, Learning Rate ∈ {0.05, 0.1, 1, 10}, Estimators ∈ {50, 100, 200}, Criterion ∈ {Friedman MSE, squared error}, and CCP-Alpha ∈ {0.0, 1, 10}.

**Table A6:** Parameters of the Gradient Boosting Classifier for the best model after grid search.

Participant	Data Preprocessing	Loss	Learning Rate	Estimators	Criterion	CCP-Alpha
1	Deletion	log-loss	0.05	50	Friedman MSE	0.0
10	Impute	log-loss	0.05	50	Friedman MSE	0.0
12	Deletion	log-loss	0.05	50	Friedman MSE	0.0
14	Preprocessed	log-loss	0.05	50	Friedman MSE	1
15	Impute	log-loss	0.05	100	Friedman MSE	10
18	Deletion	log-loss	0.05	50	Friedman MSE	0.0
19	Deletion	log-loss	0.1	200	Friedman MSE	0.0
20	Deletion	log-loss	10	100	Squared error	0.0
21	Deletion	log-loss	0.1	50	Squared error	1
23	Preprocessed	log-loss	0.05	200	Squared error	1
24	Deletion	log-loss	0.1	200	Squared error	1
26	Preprocessed	log-loss	0.05	50	Squared error	10
28	Impute	log-loss	0.05	200	Friedman MSE	1
29	Preprocessed	log-loss	0.05	50	Squared error	1

#### Appendix A.3.5. Support Vector Regressor

The grid search parameter sets are as follows. C ∈ {0.1, 1, 5, 10}, Kernel ∈ {linear, poly, rbf, sigmoid}, Degree ∈ {2, 3, 4, 5}, and Gamma ∈ {0.1, 1, scale, auto}.

**Table A7:** Parameters of the Support Vector Regressor for the best model after grid search.

Participant	Data Preprocessing	C	Kernel	Degree	Gamma
1	Preprocessed	0.1	sigmoid	0	auto
10	Impute	1	sigmoid	0	auto
12	Deletion	1	rbf	0	0.1
14	Deletion	5	rbf	0	auto
15	Deletion	0.1	sigmoid	0	auto
18	Preprocessed	5	sigmoid	0	0.1
19	Deletion	0.1	sigmoid	0	0.1
20	Deletion	0.1	poly	4	scale
21	Preprocessed	1	poly	4	scale
23	Impute	0.1	poly	2	auto
24	Impute	5	linear	0	scale
26	Preprocessed	0.1	poly	5	scale
28	Deletion	1	poly	2	scale
29	Deletion	1	poly	2	0.1

#### Appendix A.3.6. Gradient Boosting Regressor

The grid search parameter sets are as follows. Loss ∈ {squared error, absolute error, huber, quantile}, Learning Rate ∈ {0.05, 0.1, 1, 10}, Estimators ∈ {50, 100, 200}, Criterion ∈ {Friedman MSE, squared error}, and CCP-Alpha ∈ {0.0, 1, 10}

**Table A8:** Parameters of the Gradient Boosting Regressor for the best model after grid search.

Participant	Data Preprocessing	Loss	Learning Rate	Estimators	Criterion	CCP-Alpha
1	Preprocessed	Absolute error	0.05	50	Squared error	0.0
10	Preprocessed	Squared error	0.05	50	Squared error	0.0
12	Deletion	Absolute error	0.1	200	Friedman MSE	0.0
14	Impute	huber	0.1	200	Friedman MSE	0.0
15	Preprocessed	Absolute error	1	50	Squared error	10
18	Preprocessed	Absolute error	1	50	Squared error	1
19	Impute	Squared error	0.05	100	Friedman MSE	0.0
20	Deletion	Absolute error	0.05	50	Friedman MSE	1
21	Deletion	Absolute error	1	100	Squared error	10
23	Impute	Absolute error	0.1	50	Friedman MSE	10
24	Deletion	Absolute error	10	50	Friedman MSE	10
26	Preprocessed	Absolute error	1	50	Squared error	10
28	Preprocessed	Absolute error	0.1	50	Squared error	1
29	Deletion	Absolute error	1	50	Friedman MSE	0.0

#### Appendix A.3.7. Random Forest Classifier

The grid search parameter are as follows. Estimators ∈ {50, 100, 200}, Criterion ∈ {gini, entropy, log-loss}, Max Depth ∈ {None, 5, 10, 20}, Min-Samples-Split ∈ {2, 5, 10}, and Max Features ∈ {sqrt, log2, None}.

**Table A9:** Parameters of the Random Forest Classifier for the best model after grid search.

Participant	Data Preprocessing	Estimators	Criterion	Max Depth	Min-Samples-Split	Max Features
1	Preprocessed	200	log-loss	10	10	sqrt
10	Preprocessed	200	log-loss	5	2	None
12	Deletion	100	log-loss	5	2	None
14	Deletion	200	entropy	20	2	sqrt
15	Deletion	50	log-loss	20	5	log2
18	Impute	50	entropy	20	5	None
19	Deletion	50	entropy	5	2	None
20	Deletion	100	log-loss	5	5	sqrt
21	Deletion	100	log-loss	None	2	sqrt
23	Deletion	200	entropy	5	10	log2
24	Deletion	200	log-loss	20	5	None
26	Deletion	100	gini	5	5	log2
28	Deletion	200	gini	5	10	sqrt
29	Deletion	200	gini	20	2	None

#### Appendix A.3.8. Poisson Regressor

The grid search parameter sets are as follows. Alpha ∈ {0.5, 1, 2, 10}, Solver ∈ {lbfgs, Newton–Cholesky}, and Max-Iteration ∈ {100, 200, 500}.

**Table A10:** Parameters of the Poisson Regressor for the best model after grid search.

Participant	Data Preprocessing	Alpha	Solver	Max-Iteration
1	Impute	10	lbfgs	500
10	Impute	2	lbfgs	200
12	Deletion	2	lbfgs	500
14	Impute	1	Newton–Cholesky	200
15	Deletion	10	lbfgs	500
18	Preprocessed	10	Newton–Cholesky	100
19	Deletion	2	lbfgs	500
20	Deletion	10	lbfgs	500
21	Deletion	10	lbfgs	200
23	Preprocessed	10	Newton–Cholesky	500
24	Deletion	10	lbfgs	500
26	Impute	2	lbfgs	500
28	Impute	2	lbfgs	200
29	Deletion	10	Newton–Cholesky	200

#### Appendix A.3.9. Support Vector Classifier

The grid search parameter sets are as follows. C ∈ {0.1, 1, 5, 10}, Kernel ∈ {linear, poly, rbf, sigmoid}, Degree ∈ {2, 3, 4, 5}, and Gamma ∈ {0.1, 1, scale, auto}.

**Table A11:** Parameters of the Support Vector Classifier for the best model after grid search.

Participant	Data Preprocessing	C	Kernel	Degree	Gamma
1	Preprocessed	5	poly	2	auto
10	Preprocessed	10	rbf	0	0.1
12	Deletion	1	sigmoid	0	0.1
14	Deletion	0.1	linear	0	scale
15	Preprocessed	0.1	rbf	0	auto
18	Impute	0.1	poly	2	scale
19	Deletion	5	linear	0	auto
20	Deletion	1	sigmoid	0	auto
21	Preprocessed	1	poly	4	auto
23	Deletion	0.1	sigmoid	0	scale
24	Impute	10	rbf	0	scale
26	Preprocessed	0.1	rbf	0	auto
28	Preprocessed	1	rbf	0	scale
29	Deletion	5	poly	4	scale

#### Appendix A.3.10. Random Forest Regressor

The grid search parameter sets are as follows. Estimators ∈ {50, 100, 200}, Criterion ∈ {squared error, absolute error, Friedman MSE, Poisson}, Max Depth ∈ {None, 5, 10, 20}, Min-Samples-Split ∈ {2, 5, 10}, and Max Features ∈ {sqrt, log2, None}.

**Table A12:** Parameters of the Random Forest Regressor for the best model after grid search.

Participant	Data Preprocessing	Estimators	Criterion	Max Depth	Min-Samples-Split	Max Features
1	Impute	50	Absolute error	5	2	log2
10	Impute	200	Poisson	5	10	None
12	Deletion	50	Friedman MSE	10	5	log2
14	Deletion	50	Absolute error	10	5	log2
15	Preprocessed	50	Squared error	None	10	log2
18	Deletion	50	Absolute error	5	2	log2
19	Preprocessed	50	Poisson	20	2	sqrt
20	Deletion	50	Poisson	10	5	sqrt
21	Deletion	50	Absolute error	20	10	log2
23	Deletion	100	Absolute error	5	2	log2
24	Deletion	50	Absolute error	5	10	log2
26	Preprocessed	200	Absolute error	5	2	log2
28	Preprocessed	200	Absolute error	5	10	sqrt
29	Deletion	50	Friedman MSE	5	2	sqrt

### Appendix A.4. Explainability Methods

#### Appendix A.4.1. SHAP

The goal of SHAP is to explain a prediction (a single prediction) of a data instance by computing the contribution of each feature to the prediction. It uses coalitional game-theory principles to calculate how to distribute the payout among the features equitably. It computes how much each feature contributes to the model prediction for a data instance. SHAP values are a linear approximation to the Shapley values. To calculate the SHAP value for a test instance, the method replaces arbitrary combinations of features (from the test instance) with data from a background dataset. Arbitrarily changing the values of certain features and monitoring the change in model prediction gives a sense of how important a feature is, as important features will produce a more significant change in model prediction.

Thus, we obtain one SHAP value per data instance per feature. Also, the SHAP values are computed in relation to the average model prediction. Thus, a SHAP value of −0.2 for a feature in a data instance, for instance, would mean that the model prediction decreases by 0.2 from the average for a change in that feature. The background dataset is used to compute the average model prediction, and the average is computed over the entire background dataset. The SHAP values from all features combine to the difference between the prediction and the average prediction. Moreover, to compute the global feature importance for a model and a dataset, we take the mean of the absolute SHAP values for all instances in the dataset.

As we use a five-fold Cross-Validation approach, we find the SHAP values for each fold and combine them in the end. Specifically, we use the training data for each fold as the background dataset and the test dataset for each fold for the SHAP value computation. Once we have the SHAP values for the test dataset of each fold, we sum the SHAP values across folds to obtain the overall feature importance. Using the test set in five-fold Cross-Validation ensures no overlapping data instances between folds, and the SHAP values computed over all the folds cover the entire dataset.

#### Appendix A.4.2. ALE Plots

ALE plots describe how certain features influence the model prediction. ALE works well even when features are correlated by providing the individual feature effect uninfluenced by any correlated feature effects. As some degree of correlation between features can be expected, this explanation method is well suited for our dataset and models.

It finds the effect of a feature over a dataset by dividing the feature values into regions and then using the differences to calculate the average change in the prediction in the local neighbourhood (within the region). This is termed as local effects. Once the local effects across all regions have been computed, they are accumulated to obtain the ALE value. The average of all ALE values from all samples is then subtracted from the accumulated value to obtain the final ALE value. Thus, the value of the ALE can be interpreted as the main effect of the feature at a certain value compared to the average prediction of the data. For instance, an ALE estimate of −2 at $x_{i}=3$ means that when the $i^{th}$ feature has a value of three, the prediction is two less than the average prediction.

The plots show how the feature effect on the prediction varies with the value of the feature. This gives us an idea of whether a feature’s increase (or decrease) leads to a corresponding increase (or decrease) in the model prediction compared to the average prediction. We use the test dataset to compute the ALE value for each fold and find the overall ALE value by taking the mean of the ALE values obtained for the five folds.

#### Appendix A.4.3. Anchors

Anchors explain a prediction on the data instance of any black-box classification by finding a decision rule that *Anchors* the prediction sufficiently. A rule is said to Anchor a prediction if changes in other features do not affect the prediction. Anchors use perturbations of the data instance to generate local explanations for predictions of black-box models in the form of IF–THEN rules on the features. Moreover, it includes the notion of coverage, stating to which other, possibly unseen instances Anchors apply. This is achieved by generating perturbed samples around the original sample and checking the percentage of samples to which a rule applies (i.e., the percentage of samples with the same mood prediction). Finding Anchor rules is a multiarmed bandit problem, and its computation time increases as the number of features or the number of perturbation samples increase.

We use Anchors to show how specific predictions for classification models can be explained in a rule-based manner. This makes Anchors a good candidate to explain anomalous changes in mood. Depending on which fold of the test set the anomalous data instance comes from, we use randomly upsampled [74] training data of that fold as the background dataset for generating perturbations. This upsampling is necessary as the dataset is imbalanced, and an imbalanced dataset can lead to an imbalanced perturbation space, which may fail to yield Anchors (as some classes may not be represented in the perturbation space). Furthermore, to produce comprehensive rules, we take into account all features, including the neurocognitive assessment.

### Appendix A.5. Additional Results

**Table A13:** Table containing the metrics for the best base-model classification models for each participant (MAE: Mean Absolute Error, MAE STD: Mean Absolute Error standard deviation, MAPE: Mean Absolute Percentage Error in %, MAPE: Mean Absolute Percentage Error standard deviation in % and Part.: participant).

Metric	ucsd1	ucsd10	ucsd12	ucsd14	ucsd15	ucsd18	ucsd19
Test MAPE	7.972	18.911	27.901	46.644	14.296	22.048	54.468
Test MAE	0.336	0.697	0.575	0.905	0.467	0.824	1.151
Test MAE STD	0.146	0.113	0.078	0.147	0.115	0.186	0.176
Test MAPE STD	2.92	5.051	7.483	20.867	5.028	8.075	17.285
**Metric**	**ucsd20**	**ucsd21**	**ucsd23**	**ucsd24**	**ucsd26**	**ucsd28**	**ucsd29**
Test MAPE	19.758	41.876	38.243	6.736	33.008	19.71	78.578
Test MAE	0.636	1.153	0.884	0.158	1.099	0.61	1.258
Test MAE STD	0.182	0.299	0.308	0.075	0.213	0.173	0.208
test-MAPE STD	6.854	16.714	6.94	5.124	9.015	6.625	21.659

**Table A14:** Table containing the metrics for the best base-model regression models for each participant (MAE: Mean Absolute Error, MAE STD: Mean Absolute Error standard deviation, MAPE: Mean Absolute Percentage Error in %, MAPE: Mean Absolute Percentage Error standard deviation in % and Part.: participant).

Metric	ucsd1	ucsd10	ucsd12	ucsd14	ucsd15	ucsd18	ucsd19
Test MAPE	8.651	20.701	32.212	54.847	14.296	23.321	60.931
Test MAE	0.37	0.764	0.649	1.045	0.467	0.838	1.072
Test MAE STD	0.131	0.128	0.034	0.326	0.115	0.107	0.172
Test MAPE STD	2.824	4.73	5.738	29.28	5.028	7.429	19.505
**Metric**	**ucsd20**	**ucsd21**	**ucsd23**	**ucsd24**	**ucsd26**	**ucsd28**	**ucsd29**
Test MAPE	23.461	61.965	38.243	6.824	33.008	22.96	60.83
Test MAE	0.653	1.2	0.884	0.184	1.099	0.69	1.188
Test MAE STD	0.204	0.205	0.308	0.047	0.213	0.136	0.311
Test MAPE STD	13.667	8.522	6.94	2.563	9.015	7.414	12.391

**Table A15:** Table containing the metrics for the best MLP regression models for each participant (MAE: Mean Absolute Error, MAE STD: Mean Absolute Error standard deviation, MAPE: Mean Absolute Percentage Error in %, MAPE: Mean Absolute Percentage Error standard deviation in % and Part.: participant).

Metric	ucsd1	ucsd10	ucsd12	ucsd14	ucsd15	ucsd18	ucsd19
Test MAE	0.406	0.734	0.582	0.829	0.494	0.721	1.005
Test MAPE	0.096	0.202	0.223	0.362	0.131	0.187	0.481
Test MAE STD	0.086	0.101	0.063	0.327	0.05	0.112	0.176
Test MAPE STD	0.017	0.062	0.031	0.179	0.016	0.044	0.133
**Metric**	**ucsd20**	**ucsd21**	**ucsd23**	**ucsd24**	**ucsd26**	**ucsd28**	**ucsd29**
Test MAE	0.578	1.103	0.97	0.313	1.012	0.648	1.065
Test MAPE	0.199	0.531	0.391	0.222	0.334	0.211	0.591
Test MAE STD	0.144	0.216	0.304	0.074	0.272	0.12	0.19
Test MAPE STD	0.051	0.117	0.083	0.039	0.107	0.05	0.16

**Table A16:** Table containing the metrics for the best MLP classification models for each participant (MAE: Mean Absolute Error, MAE STD: Mean Absolute Error standard deviation, MAPE: Mean Absolute Percentage Error in %, MAPE: Mean Absolute Percentage Error standard deviation in % and Part.: participant).

Metric	ucsd1	ucsd10	ucsd12	ucsd14	ucsd15	ucsd18	ucsd19
Test MAE	0.295	0.715	0.532	0.933	0.522	0.638	0.989
Test MAPE	6.155	19.839	26.569	40.624	14.259	18.857	53.204
Test MAE STD	0.151	0.089	0.071	0.306	0.155	0.472	0.096
Test MAPE STD	2.729	4.426	4.386	28.323	3.418	18.541	12.124
**Metric**	**ucsd20**	**ucsd21**	**ucsd23**	**ucsd24**	**ucsd26**	**ucsd28**	**ucsd29**
Test MAE	0.455	1.201	0.914	0.125	1.053	0.562	1.03
Test MAPE	13.515	50.35	35.756	5.347	31.769	17.565	47.369
Test MAE STD	0.249	0.272	0.298	0.088	0.349	0.163	0.253
Test MAPE STD	6.165	10.689	6.494	4.933	9.414	6.398	18.945
